# Tight Blood Pressure Control in Chronic Kidney Disease

**DOI:** 10.3390/jcdd9050139

**Published:** 2022-04-30

**Authors:** Giorgio Gentile, Kathryn Mckinney, Gianpaolo Reboldi

**Affiliations:** 1 College of Medicine and Health, University of Exeter, Exeter EX1 2LU, UK; g.gentile2@exeter.ac.uk; 2 Department of Nephrology, Royal Cornwall Hospitals NHS Trust, Truro TR1 3LQ, UK; 3 Faculty of Biology, College of Letters and Science, University of Wisconsin-Madison, Madison, WI 53706, USA; kmckinney3@wisc.edu; 4 Centro di Ricerca Clinica e Traslazionale (CERICLET), Department of Medicine, University of Perugia, 06156 Perugia, Italy

**Keywords:** hypertension, chronic kidney disease, blood pressure targets, intensive blood pressure control, renal outcomes, cardiovascular outcomes

## Abstract

Hypertension affects over a billion people worldwide and is the leading cause of cardiovascular disease and premature death worldwide, as well as one of the key determinants of chronic kidney disease worldwide. People with chronic kidney disease and hypertension are at very high risk of renal outcomes, including progression to end-stage renal disease, and, even more importantly, cardiovascular outcomes. Hence, blood pressure control is crucial in reducing the human and socio-economic burden of renal and cardiovascular outcomes in those patients. However, current guidelines from hypertension and renal societies have issued different and sometimes conflicting recommendations, which risk confusing clinicians and potentially contributing to a less effective prevention of renal and cardiovascular outcomes. In this review, we critically appraise existing evidence and key international guidelines, and we finally formulate our own opinion that clinicians should aim for a blood pressure target lower than 130/80 in all patients with chronic kidney disease and hypertension, unless they are frail or with multiple comorbidities. We also advocate for an even more ambitious systolic blood pressure target lower than 120 mmHg in younger patients with a lower burden of comorbidities, to minimise their risk of renal and cardiovascular events during their lifetime.

## 1. Introduction

Hypertension affects 1.39 billion people worldwide (25% of the total adult population) and is the leading cause of cardiovascular disease, including stroke and myocardial infarction, and premature death [1]. Hypertension causes 7,500,000 premature deaths per year (12.8% of global casualties), outnumbering both diabetes (3,400,000 deaths, 3.4%) and obesity (2,800,000 deaths, 4.8%) [2]. As the rise in blood pressure (BP) with age is a universal feature of human aging, and as the global population is getting older, hypertension will likely become even more prevalent by 2040 [3].

Hypertension is also a leading cause of chronic kidney disease (CKD) through its harmful effects on kidney vasculature; in turn, worsening CKD leads to increased sympathetic tone and salt sensitivity, upregulation of the renin–angiotensin–aldosterone system (RAAS), endothelial dysfunction, and worsening arterial stiffness, eventually driving the further progression of hypertension [4]. This vicious cycle ultimately causes a progression towards end-stage renal disease (ESRD) requiring renal replacement therapy (RRT) [5], and even worse, a dramatic increase in cardiovascular (CV) morbidity and mortality [6]. For hypertensive people with stage 3 or 4 CKD (estimated glomerular filtration rate (eGFR) of 30–59 mL/min/1.73 m^2^ or 15–29 mL/min/1.73 m^2^, respectively), as defined by the Kidney Disease: Improving Global Outcomes (KDIGO) guidelines [7], the risk of dying of CV disease far exceeds the risk of developing ESRD [8].

Hence, blood pressure control is crucial to slow the progression of CKD and reduce the catastrophic socio-economic burden of CV events in this high-risk population [9,10]. Possible therapeutic strategies include the choice of certain drugs, isolated or in combination, which might theoretically provide additional reno- or cardioprotective benefits (e.g., ACE inhibitors, angiotensin receptor blockers (ARBs), calcium channel blockers (CCBs), etc.), or achieving specific BP targets, which are often tighter in the presence of significant albuminuria (albumin-to-creatinine ratio (ACR) > 70 mg/mmol) [11] or proteinuria (i.e., >0.3 g/24 h) [12]. However, there is still a considerable and heated debate regarding the ‘optimal’ blood pressure target in hypertensive patients with CKD. At one extreme of the spectrum are international societies such as the American College of Cardiology (ACC), which recommends a BP target lower than 130/80 mmHg independently of the level of proteinuria [13], or the International Society of Nephrology (ISN), with an even more ambitious systolic BP target of <120 mmHg. On the other side of the spectrum, the 2018 European Society of Cardiology/European Society of Hypertension (ESC/ESH) guideline recommends to lower systolic BP to a range of 130–139 mmHg, irrespective of proteinuria [14], and the 2021 NICE guidelines are even more cautious by advocating for a BP target lower than 140/90 in CKD patients with an ACR < 70 mg/mmol, and lower than 130/80 only in the presence of an ACR ≥ 70.

The current review will critically appraise all existing evidence used to create the different and subtly divergent guidelines for hypertension control in CKD patients and try to formulate a balanced opinion on the role of tight BP control in CKD by specifically focusing on BP targets rather than specific drug classes. However, for the sake of clarity we will also briefly consider the evidence on the specific reno- and cardioprotective role of some antihypertensive agents independently of the degree of BP control.

## 2. Current Guidelines on BP Targets in Non-Dialysis CKD Patients

Key guidelines from hypertension and renal societies are summarised in Table 1.

### 2.1. Hypertension Guidelines

Amongst the hypertension guidelines published over the last decade, the JNC-VIII guideline initially suggested a BP target <140/90 mmHg in CKD patients aged 18–69. In patients aged 70 or above, no specific recommendations were issued, apart from a generic advice that ”treatment should be individualized taking into consideration factors such as frailty, comorbidities, and albuminuria” [15]. The JNC-VIII committee issued its recommendations after reviewing three studies: the Modification of Diet in Renal Disease (MDRD) study [22], the African American Study of Kidney Disease and Hypertension (AASK) [23] and the Blood-Pressure Control for Renoprotection in Patients with Non-Diabetic Chronic Renal Disease (REIN-2) study [24].

The MDRD study [25] was designed as two separate randomised trials; the first one in 585 patients with a GFR between 25 and 55 mL/min/1.73 m^2^ (study A) and the second one in 255 patients with a GFR of 13–24 mL/min/1.73 m^2^ (study B). Although in both trials tight BP control (mean arterial pressure (MAP) ≤92 mmHg, i.e., 125/75 mmHg) did not improve the primary composite outcome of ESRD, time of doubling of serum creatinine or GFR reduction compared to standard BP control over 2.2 years of follow-up, a post-hoc analysis indicated a benefit from tight BP control in patients with baseline proteinuria levels >1 g/24 h [26]. However, this might be explained by the fact that ACEis were used much more frequently in patients randomised to a tighter BP control compared to a standard control, which might have led to greater renoprotective benefits in the subgroup of patients with higher baseline proteinuria [26].

The AASK trial [23] enrolled 1094 African Americans aged 18 to 70 years with hypertensive renal disease and no marked proteinuria and used a 3 × 2 factorial design to compare the effects of intensive (target MAP 92 mmHg) versus standard (102–107 mmHg) BP control, as well as ACEis, CCBs and β-blockers, on a composite outcome of death, ESRD or reduction in the GFR of ≥50%. Despite a significant difference in achieved BP amongst the two arms (128/78 vs. 141/85 mmHg) over 4.1 years of follow-up, tighter BP control did not reduce the risk of the primary composite outcome or the GFR slope (−2.21 vs. 1.95 mL/min/1.73 m^2^/year in the standard BP control group). During the 4.1 years of follow-up, the achieved BP averaged 128/78 mmHg in the lower BP group and 141/85 mmHg in the standard BP group. Interestingly, ACEis allowed a significant reduction in the risk of the primary outcome compared to CCBs and β-blockers. However, ramipril allowed a 22% and 38% greater reduction in the composite outcome compared to metoprolol and amlodipine, respectively.

The REIN-2 trial [24] enrolled 338 patients with non-diabetic CKD, randomised to either tight BP control (diastolic BP < 90 mm Hg) or standard BP control. Tighter BP control did not lead to any improvement in the primary outcome of time to ESRD over 3 years of follow-up (hazard ratio (HR) 1, 95% confidence interval [CI] 0.61–1.64; *p* = 0.99).

After three years from the publication of the JNC-VIII guidelines, in 2017, the ACC took a very different stance by suggesting a much more ambitious BP target of <130/80 mmHg in all hypertensive adults with CKD, regardless of the levels of proteinuria [13]. The main reason for this new position was the publication of the landmark Systolic Blood Pressure Intervention Trial (SPRINT) in 2015 [27]. In this trial, 9361 non-diabetic patients at high CV risk (previous CV event or with at least one risk factor, including smoking, dyslipidemia or CKD) were randomised to either intensive (systolic BP < 120 mmHg) or standard (<140 mmHg) BP control. The primary endpoint was a composite of myocardial infarction, other acute coronary syndromes, stroke, heart failure or CV death. At 1 year, achieved BP was 121.4 versus 136.2 mmHg in the intensive and standard BP control groups, respectively. The study was stopped earlier than anticipated (after a median follow-up time of 3.26 years) because of clear evidence of a significant benefit of intensive BP control on the primary composite outcome compared to standard BP control (HR 0.75, 95% CI 0.64–0.89, *p* < 0.001). Additionally, intensive BP control allowed a significant reduction in all-cause mortality (HR 0.73, 95% CI 0.6–0.9, *p* = 0.003). Similar benefits were observed in the subgroup of patients with stage 3 or 4 CKD (approximately 28% of the study population), which was considered as strong evidence in favour of a BP target lower than 130/80, especially because most patients with CKD die due to CV complications [8].

In 2018, the ESH/ESC issued updated guidance on BP control in CKD patients, which seems to be a compromise between the JNC-VIII and the ACC guidelines. In fact, the ESH/ESC guideline suggests that “in patients with CKD, BP should be lowered to <140/90 mmHg and towards 130/80 mmHg” [14]. In particular, the guidance recommends to lower systolic BP to a range of 130–139 mmHg in patients with diabetic or non-diabetic CKD [28,29,30]. The ESH/ESC committee acknowledged that a tighter BP control can significantly reduce all-cause mortality in CKD patients, as highlighted by a meta-analysis of Malhotra et al., which extracted mortality data from 18 trials that enrolled 15,924 CKD patients assigned to a more versus less intensive BP control [31]. Baseline mean systolic BP was 146 mmHg in both groups, but it dropped to 132 mmHg with tighter BP control versus 140 mmHg with a less intensive control. This 8-mmHg difference amongst the two groups translated into a 14% reduction in all-cause mortality with more intensive BP control (odds ratio (OR) 0.86; 95% CI, 0.76–0.97; *p* = 0.01), a finding without significant heterogeneity that was consistent across multiple subgroups. However, the committee also noted that achieved systolic BP was 132 mmHg in the “intensive” BP control group, and expressed some concerns on pursuing an even tighter control (i.e., <130/80). They mentioned that a systematic review by Upadhyay et al. failed to show any clinical benefit to the risk of death, CV events, ESRD or change in renal function with a BP target of <130/80 compared to <140/90 in non-diabetic CKD [32]. The ESC/ESH committee did not seem to acknowledge, however, that this meta-analysis also mentioned that “a lower target may be beneficial in persons with proteinuria greater than 300 to 1000 mg/day”, or the fact that only the MDRD, AASK and REIN-2 studies were included, for a total of just 2272 patients [22,23,24]. To further support the recommendation of a systolic BP target of 130–139 mmHg, the ESH/ESH guideline also mentioned a large retrospective study in 398,419 hypertensive patients (30% with diabetes), which showed that the lowest risk of ESRD and mortality was achieved by a systolic BP of 137 mmHg, whereas a clear increase in the risk of mortality was shown in patients with systolic BP lower than 120 mmHg [29]. However, observational studies are particularly prone to confounding and bias, and reverse causation might easily distort true causal relationships. Hence, in our opinion the ESC/ESH guideline has been influenced by the adoption of the “J-curve phenomenon”, which suggests that overaggressive BP control might actually increase the risk of fatal and non-fatal CV events, as well as renal complications, whenever systolic or diastolic BP is reduced below a certain threshold [33]. However, no evidence in favour of the J-curve emerged from meta-regression analyses of randomised studies, in which potential determinants of reverse causality (i.e., older age, heart failure and cancer) were equally distributed by randomisation between the treatment groups [34]. This is a frequently forgotten but important point against the clinical relevance of the J-curve phenomenon in regard to BP levels usually observed in trials and clinical practice.

All hypertension guidelines concur that either ACEis or ARBs should be first-line drugs in CKD patients regardless of diabetic status, although the ACC states that ACEis should be tried first, whereas ARBs should only be used in patients who are intolerant to ACEis.

### 2.2. Renal Guidelines

The critical appraisal of major renal guidelines published over the last decade allows the immediate identification of a clear turning point, namely the publication of the SPRINT trial in 2015 [27]. In the pre-SPRINT era, renal guidelines typically recommended a BP target of <140/90 in all CKD patients without albuminuria or proteinuria (in line with JNC-VIII recommendations, issued in 2014), whereas a tighter BP control of <130/80 mmHg was recommended in patients with moderately or severely increased albuminuria, or UPCR ≥ 150 g/g creatinine [16,17,18,19]. The recommendations issued by the older renal guidelines were based on a few prospective cohort studies [35,36], randomised clinical trials [22,23,26] and meta-analyses [32,37]. In particular, the recommendation for a BP target < 140/90 mmHg in CKD patients with no albuminuria or proteinuria seemed to be perfectly in line with the findings of a meta-analysis from the Cochrane Collaboration [37], which included four studies and more than 22,000 patients. [22,23,24,38]. Over 3.8 years of follow-up, intensive BP control (achieved BP: 128.6/78.3 mmHg) did not decrease the risk of all-cause death (relative risk [RR] 0.89, 99% CI 0.52–1.52, *p* = 0.93) or ESRD (RR 1.01, 99% CI 0.75–1.36, *p* = 0.92) compared to standard BP control (achieved BP: 139.2/84.5 mmHg). On the other hand, the recommendation for intensive BP control (<130/80) in patients with abnormal albuminuria or proteinuria mainly relied on observational data from the Multiple Risk Factor Intervention Trial (MRFIT) and the Okinawa mass screening program [35,36]. In MRFIT, BP values above 127/82 mmHg were associated with a significant increase in the risk of ESRD in 332,554 men (age: 35–57) [35]. Along the same line, BP readings above 131/79 in men and 131/78 in women significantly increased the risk of ESRD in 98,759 patients of the Okinawa mass screening program, even after adjusting for proteinuria [36]. The findings of those large cohort studies seemed to reconcile with the data from the MDRD and AASK studies [22,23,26] and the meta-analysis from Upadhyay et al., which considered a number of clinical outcomes, including all-cause death, CV death and ESRD [32]. Although tighter BP control (<125/75–130/80 mmHg) did not show any benefit on any CV or renal outcomes compared to standard BP control (<140/90), some possible benefits with a more intensive BP control were observed in the subgroup of patients with proteinuria > 300 mg/day (AASK) or 1000 mg/day (MDRD).

After the publication of the SPRINT trial in 2015 [27], which included a significant proportion of patients with stage 3 or 4 CKD (almost 30% of the study population), the renal community has started to debate the opportunity of adopting a BP target lower than 130/80 mmHg regardless of the levels of albuminuria or proteinuria, given the spectacular effectiveness of intensive versus standard BP control in reducing the risk of the primary composite outcome of myocardial infarction, other acute coronary syndromes, stroke, heart failure or CV death. Surprisingly, especially given the fact that most CKD patients will actually develop a fatal or non-fatal cardiovascular event rather than progressing to ESRD [8], the lively debate on BP targets in CKD did not translate into the rapid creation of new guidelines. For instance, the 2019 NICE guideline “Hypertension in adults: diagnosis and management” [39] did not incorporate the evidence from SPRINT, whereas readers were referred to the old 2014 NICE guideline for the general management of CKD [40]. From a “nephrological” perspective, and especially after the publication of the ACC [13] and ESC/ESH [14] guidelines, in March 2021 the UK Kidney Association, one of most influential renal associations worldwide, felt compelled to issue some urgent guidance for British clinicians, and decided to largely align to the ACC guideline [13] in recommending a BP target of <130/80 mmHg in all patients with CKD to improve CV outcomes, if tolerated by the individual and following a shared decision-making discussion with the patient [20]. In the same month (March 2021), the eagerly awaited KDIGO 2021 Clinical Practice Guideline for the management of BP in CKD was finally released [21]. In fairness, the KDIGO working group started to create a new guideline in 2017, following the Controversies Conference in Edinburgh, which was convened to “identify emerging evidence, ongoing controversies, and unsettled questions” [41], but the updated guideline was only released in 2021, partly because of the impact of the Coronavirus (COVID-19) pandemic. The 2021 KDIGO guideline is even bolder than the UK Kidney Association guideline and suggests that all adults with CKD (not receiving dialysis) and hypertension should be treated to reach a target systolic BP < 120 mmHg, if tolerated. This recommendation is largely based on the cardioprotective, survival and potential cognitive benefits of intensive BP control in the SPRINT trial and its ancillary studies, including the SPRINT MIND and a pre-specified subgroup analysis of CKD patients by Cheung et al. [27,42,43]

In August 2021, NICE finally released their updated guidance for CKD patients [11]. However, this guidance puts much emphasis on ACR values ≥70 mg/mmol to identify higher-risk CKD patients that would benefit from tighter BP control (<130/80 mmHg), whereas the “traditional” BP target of <140/90 mmHg is still recommended in people with an ACR < 70 mg/mmol.

## 3. Benefits and Harms of Specific Antihypertensive Drugs in CKD Patients

### 3.1. Non-Diabetic Kidney Disease

At each level of achieved BP, ACEis guarantee additional renoprotective benefits compared to other antihypertensive drugs that do not block the RAAS in patients with non-diabetic kidney disease. Those class-specific renoprotective benefits do not simply translate into an additional reduction in urinary protein excretion despite identical BP reduction [44], but into a significant reduction in the risk of ESRD or a combined outcome of ESRD and the doubling of serum creatinine compared to other antihypertensive drugs that do not inhibit the RAAS, as highlighted by a meta-analysis on patient-level data, which included two landmark trials [45,46,47]. However, these additional BP-independent renoprotective effects were only evident in the subgroup of patients with baseline proteinuria >500 mg/24 h (or an ACR > 30 mg/mmol). Similar findings come from another meta-analysis showing a 40% decrease in the risk of ESRD or the doubling of serum creatinine with ACEis compared to a placebo [48], and from a randomised trial enrolling non-diabetic patients with advanced CKD [49]. On the other hand, there is no clear evidence of any BP-lowering independent reno- or cardioprotective effects with ACEi treatment in patients with non-diabetic kidney disease and proteinuria lower than 500 mg/24 h [50,51,52]. Hence, ACEis are mainly beneficial in people with higher baseline levels of albuminuria or proteinuria, [53,54,55] a key point reflected in recent renal guidelines [11,21].

ARBs reduce proteinuria over the short- (1–4 months) and long-term (5 to 12 months) to a similar degree as ACE inhibitors, whereas a dual RAAS blockade with ACE inhibitors and ARBs reduces proteinuria even further compared to either agent alone [56], although this did not translate into an improvement in the GFR in a meta-analysis that included 109 patients with IgA nephropathy from six randomised controlled trials [57].

ACEis or ARBs significantly reduced a composite CV outcome of myocardial infarction, coronary revascularisation, unstable angina, stroke and other adverse cardiovascular events compared to the control treatment in patients with non-diabetic kidney disease (RR 0.56, 95% CI 0.47–0.67, *p* < 0.001), but those benefits were not evident in individual outcomes of the composite endpoint [58].

### 3.2. Diabetic Kidney Disease

Alongside intensive glycemic control [59,60,61], BP control is a cornerstone of the prevention and treatment of diabetic kidney disease. In a meta-analysis of randomised trials enrolling diabetic patients with or without CKD, ARBs significantly improved several renal outcomes, including ESRD, doubling of serum creatinine, progression from moderately increased albuminuria to severely increased albuminuria and regression from moderately increased albuminuria to normoalbuminuria, compared to a placebo. However, ARBs did not improve all-cause mortality, while ACEis decreased all-cause mortality and the progression from moderately to severely increased albuminuria, but failed to reduce the risk of ESRD or the doubling of serum creatinine [62]. A subsequent meta-analysis from Sarafidis et al. [63] reached very similar conclusions, although ACEis did not reduce the risk of all-cause mortality, likely due to the inclusion of the large Non-Insulin-Dependent Diabetes, Hypertension, Microalbuminuria or Proteinuria, Cardiovascular Events, and Ramipril (DIABHYCAR) trial, which did not show any benefit of ramipril versus a placebo on the risk of all-cause mortality [64]. However, the dose of ramipril used in the DIABHYCAR trial was really small (1.25 mg) compared to the dosage used in studies where the ACEis had much more encouraging results on all-cause mortality, including the micro-HOPE [65]. ACEis or ARBs can also reduce the risk of heart failure and other CV outcomes, including myocardial infarction and stroke, in patients with diabetic kidney disease [58]. Finally, a network meta-analysis including 119 randomised controlled trials and 64,768 CKD patients with or without diabetes showed that both ACEis and ARBs can decrease the risk of major CV events and renal failure, although only ACEis also decreased the risk of all-cause mortality compared to the active control [66].

Patients with type 2 diabetes and moderately increased albuminuria treated with higher doses of ARBs show a sustained reduction in albumin excretion rate, less progression to severely increased albuminuria and increased regression to normoalbuminuria, compared to lower doses of the same drugs, although there are no data on the effects of different doses of ARBs on all-cause mortality of CV outcomes [67]. ACEi/ARB combination treatment is not routinely recommended in diabetic kidney disease, and to the best of our knowledge, it is not endorsed by any recent hypertension or renal guidelines. The main reason for this stance is that a dual RAAS blockade (ramipril plus telmisartan), compared to monotherapy, did not decrease the risk of the primary composite outcome of all-cause death, ESRD or the doubling of serum creatinine in the Ongoing Telmisartan Alone and in Combination with Ramipril Global Endpoint Trial (ONTARGET) [68], a finding consistent with a meta-analysis from Kunz et al. [56].

## 4. Blood Pressure Targets in Special Populations

### 4.1. Children

The main evidence on BP targets in children with CKD derives from the Effect of Strict Blood Pressure Control and ACE inhibition on the Progression of CKD in Pediatric Patients (ESCAPE) trial [69], which randomised 385 children (aged 3–18) with CKD, all treated with ACEis, to either intensive BP control (target 24-h MAP below the 50th percentile) or standard BP control (MAP between the 50th and the 99th percentile), and added additional medications that did not block the RAAS. Over 5 years of follow-up, intensive BP control significantly decreased the risk of the primary composite renal endpoint of progression to ESRD or the time to 50% decline in the GFR compared to standard BP control (HR 0.65; 95% CI, 0.44 to 0.94; *p* = 0.02). Those benefits were even higher in children with higher baseline proteinuria. The findings from the ESCAPE trial have been recently appraised by the NICE committee, which agreed that the therapeutic target in children and young people with CKD and proteinuria is to keep systolic BP below the 50th percentile for their corresponding height [70]. This recommendation is also endorsed by the 2021 KDIGO guideline, which suggests that “24-h MAP measured using ambulatory blood pressure monitoring (ABPM) should be lowered to one that is at ≤50th percentile of normal children with corresponding age, sex, and height” [21].

### 4.2. Elderly

In 2021, the NICE committee critically appraised the scant evidence on specific BP targets in the elderly and concluded that none of the available data are useful for formulating a specific recommendation in those patients [70]. The committee also discussed the possible increase in adverse events potentially associated with tighter BP control in the elderly, especially dizziness and falls. Although it was noted that this concern was opinion-based rather than evidence-based, the committee agreed to cross-reference the recommendations in the 2019 NICE hypertension guideline for people who are frail or with multiple health concerns [39]. Specifically, the guideline says that “the use of clinical judgement should be highlighted in decision making for people with frailty or multimorbidity” and that “a number of factors should be considered when discussing treatment options in this group”. This advice is actually very close to the one originally included in the JNC-VIII guideline in 2014 (i.e., in patients aged 70 or above, “treatment should be individualized taking into consideration factors such as frailty, comorbidities, and albuminuria”) [15].

The 2021 KDIGO guideline supported the idea that, in most CKD patients and hypertension, the CV benefits of a target systolic BP < 120 mmHg versus <140 mmHg outweigh the risk of harm, even in the frail and elderly [21]. However, although over 40% of the 2646 CKD patients of the SPRINT trial were aged 75 or above, the median frailty index (FI) was 0.18 in the 1159 participants aged 80 or older [71]. As the FI needs to be >0.21 to classify a patient as ‘frail’, this means that the typical elderly patient enrolled in the SPRINT trial was an ambulatory patient likely to attend mostly outpatient clinical care practices, as well as the appointments for the study itself. Also, diabetic patients were excluded from SPRINT. This means that the KDIGO recommendation on the benefits of a systolic BP target <120 mmHg “even in the frail and elderly” is questionable, as only 27.6% of the SPRINT patients were actually frail [71], whereas the remaining three quarters were unlikely to represent the “typical” elderly patients seen in daily clinical practice who might well have a much higher burden of comorbidities. This is a very relevant point in our opinion, as a 1% increase in the FI is associated with increased risk for self-reported falls, injurious falls, and all-cause hospitalisations [71].

### 4.3. Dialysis Patients

In patients with ESRD undergoing haemodialysis or peritoneal dialysis, there is a staggering increase in CV morbidity and mortality, which can be 10- to 100-fold higher than in the general population [72]. Unsurprisingly, given the fact that up to 80% of dialysis patients are also hypertensives, and often with poor BP control [73], antihypertensive treatment can significantly decrease their CV morbidity and mortality, as well as all-cause mortality [74]. However, to the best of our knowledge there is a single randomised controlled trial comparing the CV benefits of different BP targets in patients on dialysis treatment, the Blood Pressure in Dialysis (BID) pilot study [75,76]. In this trial, 126 dialysis patients with a 2-week average predialysis systolic BP of ≥155 mmHg were randomised to either a predialysis SBP target of 110–140 mm Hg or 155–165 mmHg. The mean difference in systolic BP achieved between the two arms was 12.9 mmHg. At 12 months, there was no significant difference in the median change to the left ventricular mass from the baseline amongst participants assigned to intensive or standard BP control (median difference: −0.84 g/m^2^ (interquartile range, IQR: −17.1 to 10.0) in the intensive arm, versus 1.4 g/m^2^, (IQR: −11.6 to 10.4) in the standard arm, *p* = 0.43). A non-statistically significant increase in the risk of hospitalisation and vascular access thrombosis was observed in the intensive arm. Unfortunately, no major CV events were assessed in this pilot trial [77]. Although some very outdated guidelines, including the 2005 K/DOQI [78], the 2006 haemodialysis guideline from the Canadian Society of Nephrology [79] and the 2012 guideline from the Japanese Society for Dialysis Therapy [80] consistently suggest a predialysis BP target <140/90 mmHg in such patients, there is a deafening silence on the topic in all recent renal guidelines, including the 2021 KDIGO guideline, which specifically talks about “BP management in patients with CKD, with or without diabetes, not receiving dialysis” [21]. This is hardly surprising, given the lack of any evidence on the effects of intensive versus standard BP control on hard CV outcomes, including CV mortality, in dialysis patients. Hence, nephrologists are forced to rely on observational data for some guidance on the desirable BP targets in ESRD patients on dialysis treatment, but those data epidemiological findings are conflicting [81,82,83,84,85,86] as they are biased by reverse causality issues [87] or simply disregard the significant differences in comorbidities, age, ethnicity, and socio-economic status in such patients [88]. Hence, following the publication of the BID pilot study, additional high-quality randomised controlled trials assessing the effect of different BP targets on CV outcomes in dialysis patients are warranted to shed some light on this grey area of the literature.

## 5. Conclusions

Despite many years of heated debate in the hypertension and nephrology communities, the jury is still out on the desirable BP target in CKD patients. Undoubtedly, the publication of the SPRINT trial in 2015 [27] has been a seismic event to which hypertension societies [13,14] have reacted more promptly (three years earlier) than the renal ones [11,20,21]. On the other hand, the new renal guidelines issued in 2021 are in some cases as bold as the ACC guideline in suggesting a tighter BP control of <130/80 mmHg regardless of the levels of proteinuria, such as the UK Kidney Association guideline [20], and in some cases even bolder, such as the eagerly anticipated 2021 KDIGO guideline [21], which pushed the “optimal” systolic BP target in CKD patients to an unprecedented <120 mmHg. In stark contrast, the 2021 NICE guideline seems to adopt a much more conservative approach by recommending a more traditional BP target of <140/90 in CKD patients with an ACR of <70 mg/mmol and <130/80 in the presence of an ACR of ≥70 mg/mmol. Those targets are strikingly similar to pre-2015 renal guidelines [16,17,18].

Thus, what kind of BP target should be adopted by clinicians in daily clinical practice for their patients with CKD and hypertension? Our attempt to provide a balanced answer to this clinical dilemma will start from what we consider to be a critical point: in CKD patients, the risk of dying from CV disease widely exceeds the risk of developing ESRD [8]. Shockingly, a post-hoc analysis of the CV and renal outcomes in the Antihypertensive and Lipid-Lowering Treatment to Prevent Heart Attack Trial (ALLHAT) showed that 1337 out of 5545 patients with a baseline-estimated GFR lower than 60 mL/min/1.73 m^2^ died from CV disease over the extended follow-up period (4.9 years of randomised trials plus 4 years of extension), whereas only 461/5545 developed ESRD [89]. In other words, if we imagine three patients with stage 3 or 4 CKD and we follow them up over a period of 8.9 years, we will observe that two patients will die from a CV event, whereas only one will develop ESRD. Hence, our efforts should pragmatically prioritize the prevention of CV events rather than the prevention of ESRD in CKD patients, and the adoption of a pragmatical target BP should never neglect this unquestionable reality. To ascertain the benefits of more versus less intensive BP control, we recently published an updated trial sequential analysis on 16 randomised controlled trials which compared different BP targets and reported specific CV outcomes, including CV death, myocardial infarction, stroke and heart failure [90]. Our trial sequential analysis aimed at estimating whether the evidence progressively accrued on the aforementioned outcomes can be considered strong and conclusive; the logic of “early stopping rules” used during randomised controlled trials to establish whether it is still ethical to continue the study on the basis of data accrued thus far can be applied to the trial sequential analysis to understand if the accrued data are conclusive and no further randomised controlled studies are needed [91,92]. Notably, at least eight studies included in our analysis targeted a systolic BP lower than 130 mmHg and a diastolic BP lower than 80 mmHg in the more intensive arm. Our cumulative trial sequential analysis showed firm and conclusive evidence of the benefit of intensive BP control (i.e., <130/80 mmHg) for myocardial infarction, stroke, and heart failure. For CV death, the efficacy monitoring boundary was touched, but not crossed, after the inclusion of the Strategy of Blood Pressure Intervention in the Elderly Hypertensive Patients (STEP) trial [93], which means that albeit statistically significant, the benefit of intensive BP control on CV death might not be considered as conclusive at present. Our results are consistent with a recent meta-analysis of 10 randomised controlled trials by Zhang et al., which showed that intensive BP control reduces the risk of all-cause mortality, CV mortality and composite CV events in CKD patients [94].

Based on our recent trial sequential analysis and the meta-analysis from Zhang et al., we feel that clinicians should preferably follow the ACC guideline [13] and the UK Kidney Association guideline [20], which both recommend a target BP lower than 130/80 mmHg in all adults with CKD and hypertension, regardless of the level of proteinuria, provided that intensive BP control is well tolerated [20].

Although we commend the KDIGO working group for their courageous position on the systolic BP target of <120 mmHg, we are reluctant to endorse their statement that such a target should be routinely pursued “even in the frail and elderly”. In fact, the large majority of patients enrolled in the SPRINT trial were not frail [71], and we are somewhat concerned that this target can be difficult to achieve in daily clinical practice [21], especially in “real world” CKD patients who are very elderly (aged 80–85 or older), frail and with multiple comorbidities, wherein a BP target of <150/90 mmHg seems to be reasonable and in line with several international guidelines [39,95,96,97,98,99]. Indeed, CKD is often associated with resistance to antihypertensive treatment and failure to achieve the intensive BP target, as highlighted by a post-hoc analysis of the SPRINT study [99]. On the other hand, we also feel that clinicians should not be excessively concerned about the so-called “J-curve phenomenon” if they decide to pursue this more ambitious target in younger patients with a longer life expectancy with the objective of preventing major CV events and CV deaths. In fact, post-hoc analyses of the ONTARGET trial [100] and the Valsartan Antihypertensive Long-Term Use Evaluation trial [101] did not show any evidence of an excess risk of CV events at lower-achieved BP values, suggesting that the J-curve phenomenon is mainly explained by reverse causality due to coexisting diseases (e.g., cancer, heart failure, etc.) associated with low BP and poor outcome.

In terms of first-line drugs for CKD patients, current hypertension and renal guidelines consistently recommend the usage of either ACEis or ARBs in both diabetic and non- diabetic kidney disease for their clear reno- and cardiovascular benefits [66,102,103,104,105,106,107,108], and a full dose should be used whenever possible and well tolerated. However, clinicians should bear in mind that the majority of CKD patients will require a combination treatment with two or more antihypertensive agents to achieve a satisfactory degree of BP control (i.e., at least <130/80 mmHg if tolerated), and that additional renoprotective drugs are available, including third-generation dihydropyridine CCBs [108,109,110]. An ACEi/ARB combination treatment is not recommended by any available guideline and should be avoided because of the increased risk of acute kidney injury and hyperkalaemia. Combination treatments (e.g., ACEi or ARB plus CCB) should be tailored to the individual patient, trying to balance the need for an intensive BP control, the risk of side effects and the need to ensure long-term adherence, and concomitant CV risk factors (smoking, dyslipidemia, diabetes, etc.) should be aggressively tackled to further reduce CV and renal outcomes.

In conclusion, we recommend that clinicians pursue a target BP of <130/80 mmHg in all CKD patients with hypertension, unless they are frail (36-item FI > 0.21) [71], or very elderly (aged 80–85 or older) and with multiple comorbidities (e.g., heart failure, cancer, malnutrition, chronic infections, etc.) [111]. We also advocate for a more ambitious systolic BP target of <120 mmHg, as per the 2021 KDIGO guideline [21], in younger patients with a lower burden of comorbidities to minimise CV morbidity and mortality over their lifetime and obtain longer survival times, while avoiding unnecessary excess risk of adverse events with overaggressive BP control in very elderly patients with a significant number of comorbidities and a lower residual life expectancy.

## Figures and Tables

**Table 1:** Current guidelines on BP targets in non-dialysis CKD patients.

Guideline Agency	Country	Year	Target Recommendation (mmHg)	First-Line Agents Recommended
*Hypertension societies*				
Joint National Commission on Prevention, Detection, Assessment and Treatment of Hypertension (JNC-VIII) [15]	United States	2014	<140/90 (people aged 18–69 with CKD or diabetes) No recommendation for CKD patients aged 70 or above (“treatment should be individualized taking into consideration factors such as frailty, comorbidities, and albuminuria”)	ACEi or ARB (regardless of ethnicity or diabetic status)
American College of Cardiology (ACC) [13]	United States	2017	<130/80 in all adults with hypertension and CKD, regardless of proteinuria	ACEi (ARB if the ACEi is not tolerated)
European Society of Hypertension/European Society of Cardiology (ESH/ESC) [14]	Europe	2018	Systolic BP between 130 and 139	ACEi or ARB (regardless of diabetic status)
*Renal societies*				
European Best Practice Guidelines (EBPG) [16]	Europe	2013	<140/90 (no albuminuria/proteinuria) <130/80 (ACR ≥ 30 mg/g, i.e., at least moderately increased albuminuria, or UPCR ≥ 150)	ACEi or ARB
Italian Society of Nephrology [17]	Italy	2013	<140/90 (normoalbuminuria) <130/80 (albuminuria >30 mg/24 h, i.e., at least moderately increased albuminuria)	ACEi or ARB
Kidney Health Australia- Caring for Australasians with Renal Impairment (KHA-CARI) [18]	Australia	2014	<140/90 (normoalbuminuria or moderately increased albuminuria) <130/80 (severely increased albuminuria)	ACEi or ARB
Canadian Society of Nephrology (CSN) [19]	Canada	2015	<140/90 (regardless of diabetes or proteinuria)	ACEi or ARB
UK Kidney Association (UKKA) [20]	UK	2021	<130/80 (if, following a shared decision-making discussion, it is tolerated by the individual)	No explicit recommendation
National Institute for Health and Care Excellence (NICE) [11]	UK	2021	<140/90 (if ACR < 70 mg/mmol) <130/80 (if ACR ≥ 70 mg/mmol; target range 120 to 129 mmHg)	ACEi or ARB (titrated at the highest tolerated dose, for any patient with ACR > 30 mg/mmol)
Kidney Disease: Improving Global Outcomes (KDIGO) [21]	Global (International Society of Nephrology)	2021	Systolic BP <120 (if tolerated)	ACEi or ARB (for any patient with moderately or severely increased albuminuria)

Abbreviations: ACEi—Angiotensin converting enzyme inhibitor; ARB—angiotensin receptor blocker; ACR—albumin-to-creatinine ratio; UPCR—urine protein-to-creatinine ratio; UK—United Kingdom.

## Data Availability

Not applicable.
